# A comparison of the characteristics of iOS and Android users of a smoking cessation app

**DOI:** 10.1007/s13142-016-0455-z

**Published:** 2017-02-06

**Authors:** Harveen Kaur Ubhi, Daniel Kotz, Susan Michie, Onno C. P. van Schayck, Robert West

**Affiliations:** 1 Cancer Research UK Health Behaviour Research Centre, Department of Epidemiology and Public Health, University College London, 1-19 Torrington Place, London, WC1E 6BT UK; 2 Institute of General Practice, Medical Faculty of the Heinrich-Heine-University Düsseldorf, Düsseldorf, Germany; 3 Department of Family Medicine, CAPHRI School for Public Health and Primary Care, Maastricht University Medical Centre, Maastricht, The Netherlands; 4 Department of Clinical Effectiveness and Health Psychology, Centre for Outcome Research and Effectiveness, University College London, 1-19 Torrington Place, London, WC1E 6BT UK

**Keywords:** Smoking cessation, Mobile, Smartphone, Apps, Characteristics, SF28, iOS, Android

## Abstract

iOS and Android smartphone users may differ in ways that affect their use and likelihood of success when using a smoking cessation application (app). If so, it may be necessary to take the device type (iOS and Android) into account when designing smoking cessation apps and in studies evaluating app effectiveness. How do socio-demographic and smoking characteristics, potentially relevant to engagement and cessation outcomes, of the SF28 app users differ between those using the iOS version and those using the Android version? Data were collected between October 2013 and April 2015. The variables measured were age, gender, social grade, time since the most recent quit attempt, choice of medication use (nicotine replacement therapy or varenicline), weekly expenditure on cigarettes, cigarettes smoked per day, reason for using the app and quit date set. The alpha was set to *p* < 0.006 to adjust for multiple comparisons. A total of 1368 users were included in the analysis. iOS and Android device users were similar in terms of age, social grade, weekly expenditure on cigarettes and cigarettes smoked per day. Compared with Android users, iOS users were more likely to have downloaded the app for a serious quit attempt (74.3 versus 69.6%, *p* = 0.001), made a quit attempt within the last 12 months (59.6 versus 45.9%, *p* < 0.001) and set their quit date on the day of registration (61 versus 46.2%, *p* < 0.001). They were less likely to have used stop-smoking medication to support their quit attempt (31.5 versus 48.6%, *p* < 0.001). Differences between smokers using the iOS version of smoking cessation apps and those using the Android version may influence quit success.

## INTRODUCTION

Smartphone applications (hereinafter referred to as ‘apps’) might aid smoking cessation [1, 2]. Currently, the most commonly used operating systems for smartphones are Apple’s iOS and Google’s Android [3]. iOS and Android smartphone users differ in socio-demographic characteristics [4] and in their usage of apps [5]. If these or other differences are found in users of smoking cessation apps, they might influence the chances of a successful quit attempt. In that case, the device type (iOS and Android) would need to be routinely used as a covariate in studies evaluating app effectiveness. Currently, there is no published information regarding this topic. This study aimed to address this gap in the literature.

In terms of ways in which iOS and Android users have been found to differ in general, iOS users are more likely to be women, to be in their mid-30s, to have a graduate degree, to belong to a higher income group and to be more knowledgeable about technology than Android users [4]. Any of these factors may influence engagement with apps of different types. It is not known whether this general pattern of difference is reflected in smokers using smoking cessation apps. Differences between iOS and Android users may influence or reflect one or more of the three pillars of behaviour: capability, opportunity and motivation [6, 7]. For example, in contrast to novice smartphone users, advanced users may influence the capability to engage with complex app features (these users generally seek to understand the app’s mechanics and how it works). Gender and age may influence the aesthetic appeal of different features and therefore the motivation to use the app. Hence, these interactions may influence a user’s motivation to engage with apps. Although there is limited research on utilising a user-centred approach in terms of feature preferences by gender, there is extensive literature explaining women’s aesthetic responses to properties, including colour, symmetry and harmony [8–11]. These properties could serve as a reference to consider how female users could interact with digital interfaces. In addition, not all apps work equally well on Android devices; this may be due to diverse device manufacturers (Samsung, HTC, LG, etc.), varying screen sizes or different variants of Android operating systems. These factors may influence the way in which users engage with particular app features (opportunities).

Turning to user characteristics that may influence smoking cessation outcomes whether or not these have been associated in general with iOS versus Android users, higher educational level and income have been found to be associated with higher chances of quitting smoking [12]. In addition, a number of smoking-related variables have been found to be predictive of quit success: cigarettes smoked per day [13–17], the use of stop-smoking medication [18], time since the most recent quit attempt [18–20] and whether a quit attempt is made immediately or planned in advance [13, 14]. It is not clear whether, or in what ways, smokers who use iOS and Android apps differ in these characteristics.

**Fig. 1:**
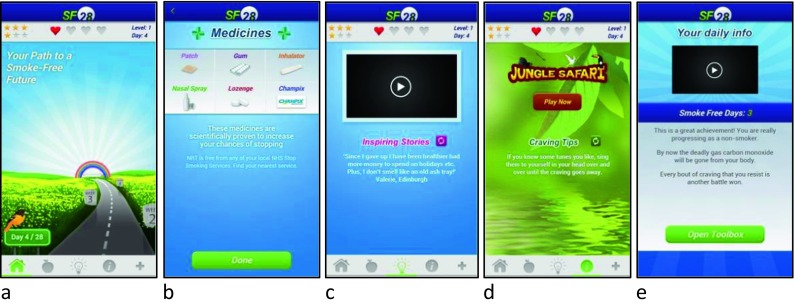
Screenshots of the SF28 app. **a** Homescreen of the SF28 app. **b** App advising about the use of stop-smoking medication. **c** App includes inspirational statements to bolster positive motivation. **d** App advising on how to help cope with cravings when they occur (inbuilt distraction game). **e** App advising on how to make changes to lifestyle to reduce exposure to smoking triggers

To study the differences in users’ characteristics, this study used a smoking cessation app, SF28, (short for SmokeFree28), which is free to download in the Apple App Store for iOS users and in the Google Play Store for Android users [21]. This app focuses on behaviour change techniques that, from theory [22] and evidence [23], would be expected to aid smoking cessation. The app used the PRIME theory of motivation (Plans, Responses, Impulses, Motives and Evaluations) [24] and prior evidence on effective behaviour change techniques for smoking cessation as the basis for its design. From PRIME theory, the app aimed to address multiple levels of motivation (planning, evaluative beliefs, desires, and immediate impulses and inhibitions). SF28 involves setting a highly salient target of becoming 28-days smoke-free and monitoring progress towards that target. Using a ‘toolkit’ of activities and aids, the app provides behavioural counselling to smokers trying to quit. The toolkit includes: (a) advice about the use of stop-smoking medication; (b) advice on changes to lifestyle in order to reduce exposure to smoking triggers; (c) inspirational statements from smokers who have stopped smoking to bolster positive motivation; and (d) advice to help cope with cravings when they occur (Fig. 1) [1]. The app is the same in terms of its design and content for both the iOS and Android versions [21].

The research question addressed by this study was the following: How do socio-demographic and smoking characteristics, potentially relevant to engagement and cessation outcomes, of the SF28 app users differ between those using the iOS version and those using the Android version?

## METHODS

### Study design

This was a comparative observational study involving anonymised data automatically collected from SF28 users between October 2013 and April 2015. In the initial text on sign-up, users were made aware that anonymised data would be used for research. Ethical approval was granted by the University College London Ethics Committee.

### Participants

A total of 1937 registrations were recorded. Participants were included in the analysis if they were cigarette smokers, aged 18 or over at the time of registration and had set a quit date within 2 weeks of downloading the app. The core objective of the SF28 app is to support smokers through their quit attempt rather than help them reduce their daily cigarette consumption or prepare them for their quit attempt. App users who registered more than once were excluded from the study.

### Data collection

Once a potential user downloaded the SF28 app onto their mobile device, the app asked permission for his or her data to be collected and used for research purposes. Each time a user logged in, SF28 routinely collected data from the session and uploaded it to a secure server. No identifying information was collected apart from the device identification number (to identify multiple registrations). SF28 users were encouraged to open the app each day for at least 28 days from their target quit date. The app continues to be available after that time but its content does not change after 28 days. The app can be found by searching for stop-smoking apps in the Apple App Store for iOS users and in the Google Play Store for Android users. No promotion was undertaken so the usage depended on iPhone and Android users finding it through searches or via word of mouth.

At the time of initial registration, the SF28 app assessed the following: age, gender, social grade (manual, non-manual or other), time since the most recent quit attempt (no previous quit attempt, over a year ago or in the last year), choice of medication use (none, nicotine replacement therapy or varenicline), weekly expenditure on cigarettes (£s per week), number of cigarettes smoked per day, reason for using the app (‘I plan to make a serious quit attempt’, ‘I am interested in quitting but just not yet’, ‘I am just testing the app’ or ‘other’) and quit date set (on the day of registration, in future from the day of registration or before the day of registration). For medication use, we did not include bupropion as an option because its use in England is very rare [25]. In our study, the non-manual group is similar to what in other countries might be termed as ‘white collar’ and the manual group as ‘blue collar’. We kept the response to just these categories in oder to minimise the response burden.

In addition, the app collected information on whether the current app, SF28, was downloaded with the intention of making a serious quit attempt. Currently, the proportion of app users signing up with the intention of quitting seriously is not known, and this variable could potentially impact the success of a quit attempt. For any given user, a serious quit attempt was described as an attempt to give up smoking altogether. User engagement was measured by recording the number of times users opened the SF28 app.

At the time of initial registration with the SF28 app, it would also be useful to capture some additional information on variables that are associated with improving the chances of a successful quit attempt (such as smoking restrictions at home and cigarette dependence). Technology literature indicates that a large majority of users will abandon an app within the first minute if onboarding (i.e. the process of walking a user through a few screens to orient them with an app and its features) involves filling out lengthy registration forms [26]. Providing a simple onboarding experience to app users can significantly help to reduce abandonment rates [26]. Therefore, it was judged that the questions asked struck a reasonable compromise between the information gathered and the need to acquire users.

### Analysis

Data were analysed using SPSS 21.0. To compare the characteristics between the two groups (iOS and Android) of users, we conducted analysis of variance (ANOVA) for continuous variables and chi-squared tests for categorical variables. Bonferroni correction was used to account for multiple comparisons, so the alpha value was set to *p* < 0.006 for the nine comparisons involving the full sample and for the eight comparisons for those making a serious quit attempt. This is considered conservative for correlated *p* values as is the case in this study.

## RESULTS

A total of 1368 users were included in the analysis. Of these, 1049 (76.7%) used an iOS device and 319 (23.3%) used an Android device (Table 1). In all, 1001 (73.2%) users downloaded SF28 for a serious quit attempt.

**Table 1:** Socio-demographic and smoking characteristics of SF28: iOS and Android users

Characteristics	All users *n* = 1368	iOS users *n* = 1049	Android users *n* = 319	*p* value
Age (in years), mean (SD)	33.2 (9.6)	33 (9.4)	33.7 (10.2)	*p* = 0.257
Weekly spend on cigarettes (in £s), mean (SD)	35.9 (24.1)	36.1 (24.1)	35.2 (24.1)	*p* = 0.559
Cigarettes smoked per day, mean (SD)	16.5 (7.8)	16.5 (7.5)	16.4 (8.7)	*p* = 0.925
Women, % (*n*)	63.9 (858)	65.8 (679)	57.7 (179)	*p* = 0.01
Social grade, % (*n*)				
Manual	31.6 (337)	30.8 (252)	34.1 (85)	*p* = 0.089
Non-manual	37.2 (397)	39 (319)	31.3 (78)	
Other	31.2 (333)	30.2 (247)	34.5 (86)	
Time since the most recent quit attempt, % (*n*)				
No previous quit attempt	14.8 (176)	14.5 (132)	15.5 (44)	*p* < 0.001
Over a year ago	28.9 (344)	25.9 (235)	38.5 (109)	
In the last year	56.3 (671)	59.6 (541)	45.9 (130)	
Choice of medication, % (*n*)				
No medication	64.5 (882)	68.4 (718)	51.4 (164)	*p* < 0.001
Nicotine replacement therapy (NRT)	25.3 (346)	25.7 (270)	23.8 (76)	
Varenicline	10.2 (140)	5.8 (61)	24.8 (79)	
Quit date set, % (*n*)				
On the day of registration	57.5 (748)	61 (607)	46.2 (141)	*p* < 0.001
In future from the day of registration	42.4 (551)	38.9 (387)	53.8 (164)	
Before the day of registration	0.1 (1)	0.1 (1)	0 (0)	
Reason for using the app, % (*n*)				
I plan to make a serious quit attempt	73.2 (1001)	74.3 (779)	69.6 (222)	*p* = 0.001
I am interested in quitting, but not yet	13.5 (184)	14.3 (150)	10.7 (34)	
I am just testing the app	10.5 (143)	8.9 (93)	15.7 (50)	
Other	2.9 (40)	2.6 (27)	4.1 (13)	

Missing data: gender *n* = 26, social grade *n* = 301, time since the most recent quit attempt *n* = 177 and quit date set *n* = 68

Compared with Android users, iOS users were more likely to download the app for a serious quit attempt (iOS = 74.3%; Android = 69.6%; *χ* ^2^ = 15.87, df = 3, *n* = 1368, *p* = 0.001), had made a quit attempt within the last 12 months (iOS = 59.6%; Android = 45.9%; *χ* ^2^ = 19.2, df = 2, *n* = 1191, *p* < 0.001) and had set their  quit date to the same when they downloaded the app (iOS = 61%; Android = 46.2%; *χ* ^2^ = 21.35, df = 2, *n* = 1300, *p* < 0.001). Android users were more likely to use stop-smoking medication to quit smoking as compared with iOS users (Android = 48.6%; iOS = 31.5%; *χ* ^2^ = 97.2, df = 2, *n* = 1368, *p* < 0.001, NRT *p* = 0.789, no medication *p* < 0.001 and varenicline *p* < 0.001). However, no clear difference in occupational group (manual group: iOS = 30.8%; Android = 34.1%; *χ* ^2^ = 4.84, df = 2, *n* = 1067, *p* = 0.089) emerged among smokers in relation to the type of device used (Table 1).

As determined by analysis of variance, the iOS and Android users were similar in terms of age (iOS users 33 years (SD 9.4) and Android users 33.7 years (SD 10.2)) (*F*(1, 1366) = 1.28, *p* = 0.257), weekly spend on cigarettes (iOS users £36.1 (SD 24.1) and Android users £35.2 (SD 24.1)) (*F*(1, 1366) = 0.34, *p* = 0.559) and cigarettes smoked per day (iOS users 16.5 (SD 7.5) and Android users 16.4 (SD 8.7)) (*F*(1, 1366) = 0.009, *p* = 0.925). The mean number of logins made on the SF28 app was 4.6 (SD 9.6) for iOS users and 4.9 (SD 6.9) for Android users (*F*(1, 1366) = 0.38, *p* = 0.536) (Table 1).

The characteristics of users who downloaded the app with the intention of making a serious quit attempt were found to be similar to the  characteristics of the full sample (Table 2). The mean number of logins made on the SF28 app was 4.8 (SD 9.6) and 5.2 (SD 7) respectively for iOS and Android users who downloaded the app with the intention of making a serious quit attempt (*F*(1, 999) = 0.33, *p* = 0.568).

**Table 2:** Socio-demographic and smoking characteristics of serious SF28 quitters (“I plan to make a serious quit attempt”): iOS and Android users

Characteristics	Users making a serious quit attempt *n* = 1001	iOS users *n* = 779	Android users *n* = 222	*p* value
Age (in years), mean (SD)	33.4 (9.5)	33.3 (9.3)	34.1 (10.3)	*p* = 0.238
Weekly spend on cigarettes (in £s), mean (SD)	36.4 (23.7)	36.3 (23.8)	36.9 (23.6)	*p* = 0.753
Cigarettes smoked per day, mean (SD)	16.3 (7.5)	16.4 (7.4)	16.3 (8.0)	*p* = 0.839
Women, % (*n*)	66.9 (660)	68.8 (529)	60.1(131)	*p* = 0.016
Social grade, % (*n*)				
Manual	31.3 (249)	30.7 (190)	33.5 (59)	*p* = 0.102
Non-manual	38 (302)	39.9 (247)	31.2 (55)	
Other	30.7 (244)	29.4 (182)	35.2 (62)	
Time since the most recent quit attempt, % (*n*)				
No previous quit attempt	10.8 (96)	10.6 (73)	11.6 (23)	*p* < 0.001
Over a year ago	28.8 (255)	25.6 (176)	39.9 (79)	
In the last year	60.4 (535)	63.8 (439)	48.5 (96)	
Choice of medication, % (*n*)				
No medication	63.3 (634)	67.9 (529)	47.3 (105)	*p* < 0.001
Nicotine replacement therapy (NRT)	26.8 (268)	27.1 (211)	25.7 (57)	
Varenicline	9.9 (99)	5 (39)	27 (60)	
Quit date set, % (*n*)				
On the day of registration	61.3 (592)	65.6 (493)	46.5 (99)	*p* < 0.001
In future from the day of registration	38.7 (373)	34.4 (259)	53.5 (114)	
Before the day of registration	0 (0)	0 (0)	0 (0)	

Missing data: gender *n* = 14, social grade *n* = 206, time since the most recent quit attempt *n* = 115 and quit date set *n* = 36

## DISCUSSION

Smokers using the SF28 app on iOS devices differed from those using it on Android devices with regard to the number of characteristics that may influence their chances of quitting smoking. Compared with Android users, iOS users were more likely to download the app for a serious quit attempt, to have set their quit date to the same day when they downloaded the app and to have previously made at least one quit attempt in the last 12 months. They were less likely to use stop-smoking medication to support their quit attempt. iOS and Android users were similar with regard to age, social grade, weekly expenditure on cigarettes and number of cigarettes smoked daily.

The finding that approximately a quarter of smokers who downloaded the app did not intend to make a serious quit attempt means that this variable should be recorded in all future studies when evaluating app effectiveness. Unlike engagement with face-to-face stop-smoking support, apps have a very low threshold for initial exposure and therefore it cannot be assumed that because a user has set a quit date, he or she is making a serious quit attempt.

The pattern of difference between iOS and Android users suggests that iOS users may have a higher motivation to quit. Despite this, the usage patterns for iOS and Android users were similar. To date, research on the digital divide between iOS and Android smartphone users has assumed that these users differ in socio-demographic characteristics and in the usage of apps [4, 5]. As we did not find major socio-demographic differences between these two groups of users, it may be that the digital divide among smoking cessation app users is mainly driven by endogenous factors (intrinsic motivation, self-efficacy, etc.) rather than by exogenous factors (social grade). If this pattern is replicated in other app-based smoking cessation studies, it could be useful to assess whether this is restricted to smoking cessation apps or whether it generalises to other health apps. Improving engagement with these types of apps remains a major challenge.

Our findings suggest that the device type (iOS or Android) should be routinely used as a covariate in analyses when comparing the effectiveness of apps and when conducting meta-regressions. Moreover, there may be merit in tailoring apps for different devices according to the types of smokers who are likely to use them. The information provided in the study can be used to help achieve this objective. For example, in this study, the lower rate of medication use was observed within iOS users as compared with Android users (iOS users: 31.5% versus Android users: 48.6%). This difference  was mainly driven by prescription medication suggesting that there may be merits in tailoring apps for iOS and Android smartphone users – placing greater emphasis on iOS users using stop-smoking medication and thereby increasing the chances of quitting smoking successfully.

In this study, iOS and Android users were similar with regard to age (young), social grade, weekly expenditure on cigarettes and number of cigarettes smoked per day (dependent smokers). These findings suggest that app-based smoking cessation interventions may offer a means of equitable treatment delivery to smokers, particularly for those smokers who are from deprived socio-economic groups. Research has found that disadvantaged smokers want to stop as much as other smokers but find it more difficult [27]. Moreover, in recent times, smartphone use by younger people has grown rapidly and app-based interventions could act as an effective channel for reaching, and engaging with, adolescent and younger smokers who may not want to use traditional support channels for smoking cessation (such as face-to-face and/or group-based interventions). Furthermore, these novel smartphone-based interventions could also act as a potential alternative to break structural and cultural barriers to the adoption of smoking cessation services.

One limitation of this study is that it used data from a single app and so the findings may not generalise to apps in general. However, this study provides insights into how the digital divide between iOS and Android users that could be mainly driven by endogenous factors. Another limitation is that we did not have reliable data on smoking cessation rates for iOS and Android users; therefore, we were unable to determine whether users of one platform performed better than users of the other platform in terms of achieving the goal of being smoke-free.

To conclude, this study found some potentially important differences–which may be reflective of a motivation to quit–between smokers using the iOS version of a smoking cessation app and those using the Android version. In addition, a substantial proportion of users using the app were making a serious quit attempt. Therefore, for evaluation of smoking cessation apps, the type of smartphone device used and the quit smoking intent are important variables to take into account.  
